# Immune-modulating effects of dietary cannabinoid-receptor 2 agonist beta-caryophyllene and cooling frequency in dairy cows under environmental heat load

**DOI:** 10.3389/fimmu.2026.1931588

**Published:** 2026-09-30

**Authors:** Liam Hubner, Priscila dos S. Silva, Yana Butenko, Ben Shattenstein, Yuri Portnick, Tali Dogot, Lir Marcus, Ahmed Nasser, Mohamed Samara, Barry J. Bradford, Maya Zachut

**Affiliations:** 1 Department of Ruminant Science, Institute of Animal Sciences, Agricultural Research Organization, Volcani Institute, Rishon LeZion, Israel; 2 Department of Animal Science, The Robert H. Smith Faculty of Agriculture, Food and Environment, The Hebrew University of Jerusalem, Rehovot, Israel; 3 Intra-Institutional Services Unit, Volcani Institute, Rishon LeZion, Israel; 4 Department of Animal Science, Michigan State University, East Lansing, MI, United States

**Keywords:** cannabinoid, dairy cow, heat stress, immune cells, inflammation, β-caryophyllene

## Abstract

**Background:**

Environmental heat load negatively affects productivity, welfare and immune function in livestock. The endocannabinoid system regulates immune function, and activation of the cannabinoid receptor 2 (CB2) has mostly anti-inflammatory effects. We hypothesized that increased cooling frequency and providing a dietary CB2 agonist, β-caryophyllene (BCP), could affect immune function in heat-stressed dairy cows.

**Methods:**

Forty mid-lactation Holstein dairy cows exposed to environmental heat load conditions were assigned randomly according to their milk production, body weight, lactation number and days in lactation to one of 4 treatments: Either controls (CTL) or supplemented with BCP (at 5 mg/kg BW), and half of the cows from each dietary treatment were cooled with either 3 or 6 cooling sessions (CS) a day, creating a 2 × 2 factorial design (*n* = 10 in each group): CTL-6CS, CTL-3CS, BCP-6CS, and BCP-3CS. Milk production, feed intake and behavior parameters were measured. At week 5, six cows from each group were challenged with intra-mammary lipopolysaccharide (LPS). Vaginal temperatures and blood samples were taken for stress and immune function parameters. Data were analyzed for main effects of BCP, CS, time and their interactions.

**Results:**

BCP supplementation increased plasma BCP concentrations vs. CTL. Before the LPS-challenge, production and feed intake were not affected by BCP, but BCP increased the duration of resting bouts and reduced eating time. 6CS-cows had longer rumination time and lower panting time and respiratory rate compared to 3CS. During the LPS-challenge, BCP-cows exhibited lower vaginal temperatures during peak fever, with lowest temperatures in BCP-6CS cows. Plasma cortisol area under the curve (AUC) tended to be lower in BCP-6CS than in CTL-3CS. BCP-cows had lower blood monocyte percentages post-LPS compared to CTL; while mean platelet volume, mass and mass-distribution-width were increased in BCP vs. CTL.

**Conclusions:**

Dietary BCP during heat load lowered peak fever response to inflammatory-challenge and had moderate and specific immune-modulating and stress-mitigating effects, with some added effects with increased CS frequency. Thus, dietary supplementation with a CB2 agonist can be a novel strategy to improve immune function, stress response and welfare of dairy cows.

## 1 Introduction

Heat load, a combination of high ambient temperature and humidity, is a major abiotic factor that negatively impacts livestock productivity (1). The temperature–humidity index (THI) is a commonly used measure to indicate the degree of heat load in dairy cattle, where values of 70 or less are considered comfortable, 75–78 stressful, and values above 78 cause extreme distress and difficulty regulating body temperature (1). In the upcoming decades, climate change is expected to increase the intensity and frequency of heat load events (2). As a result, livestock, and particularly high yielding dairy cows, will be exposed to increasing levels of heat load, which will affect their welfare and productivity. Environmental heat load also affects the immune function of dairy cows, as it can initiate the inflammatory response and the immune cascade. Heat stress dysregulates immune function by increasing the expression of pro-inflammatory cytokines in blood cells of cattle, and is associated with reduced lymphocyte proliferation (3, 4). In an *in vitro* study (5), bovine peripheral blood mononuclear cells (PBMC) exposed to heat stress had increased expression of heat shock protein (HSP) 72, and reduced DNA synthesis compared to cells in normothermia. Thus, bovine immune cells react to elevated temperature directly with a loss of function, which is consistent with a reduction in immune function *in vivo*, as immune cells exposed to high temperatures show a reduction in proliferation, phagocytosis and antibody production, with higher rates of apoptosis (6). The impaired immune function during heat load increases the susceptibility to intramammary infections: heat-stressed cows were shown to exhibit reduced neutrophil phagocytic capacity, elevated somatic cell counts (SCC), and higher rates of mastitis during summer months (6). To date, the most widely used management tool to reduce the impact of heat load in dairy cows is by performing cooling sessions (CS), a combination of water soakers and fans. However, using high-throughput metrological and production data, it was recently demonstrated that management adaptation strategies, even those at the technological frontier, may be insufficient to address climate change damages to dairy cows’ production (7).

Therefore, there is a need for new strategies to improve immune function of heat stressed livestock, which would ultimately have beneficial effects on their health, productivity and welfare. The main hypothesis of the present work is that activation of the endocannabinoid system (ECS) could improve immune function in dairy cows. The ECS is a physiological-endocrine system that regulates metabolism, immune system function and the stress response in all mammals (8). The ECS includes two main canonical receptors, the cannabinoid receptor 1 (CB1) and cannabinoid receptor 2 (CB2), as well as the endogenous ligands the endocannabinoids (eCB), and enzymes that synthesize and degrade eCB (8). The eCB are lipid derivates of fatty acids (FA), synthesized in response to a stimulus such as an increase in intracellular calcium influx, metabolic stress, or cellular damage (9, 10). Our research group was among the first to characterize the ECS in dairy cows (8, 10, 11). Specifically, we have demonstrated that dietary supplementation can affect ECS activation in various tissues, and may affect immune function in dairy cows (12, 13).

The CB2 receptor is expressed highly in immune cells in mammals (8), and its activation triggers mostly an anti-inflammatory responses (14). The CB2 regulates pro-inflammatory cytokine release and plays a crucial role in controlling inflammation, including modulating immune cell migration and proliferation (15). Activation of CB2 primarily modulates immune cell responses (16), and both *in vitro* and *in vivo* studies in humans and rodents suggest that CB2 activation suppresses immune responses (15). Specifically, CB2 activation in monocytes and macrophages has been shown to decrease the secretion of pro-inflammatory cytokines like tumor necrosis factor alpha (TNFα), interleukin (IL) 1β, and IL-6 (17, 18), while simultaneously increasing the production of the anti-inflammatory cytokine IL-10 (18). Therefore, activation of CB2 could have beneficial effects on immune function in dairy cows (8).

Phyto-cannabinoids are secondary plant metabolites that are active in mammals after ingestion (19), and they interact with the cannabinoid receptors (20). The cannabinoid β-caryophyllene (BCP) is found in many botanical oils, including black pepper, oregano, cinnamon and cannabis, and it is a selectively binding agonist to the CB2 receptor (21). When BCP binds to CB2, it activates a G-coupled receptor inhibition pathway, which inhibits the production of cyclic adenosine monophosphate, thereby leading to a reduction in the activity of transcription factors (21). In rodents, activation of CB2 by BCP leads to a decrease in the expression of the pro-inflammatory cytokines TNFα and IL-1β following exposure to lipopolysaccharide (LPS), and inhibits phosphorylation of the transcription factors extracellular signal-regulated kinases 1/2 and c-Jun N-terminal kinases 1/2 in monocytes, which are associated with the expression of inflammatory genes (21). In addition, BCP at a dose of 5 mg/kg body weight reduces edema caused by carrageenan injection in wild-type mice, but not in mice lacking the CB2 receptor, suggesting that BCP has anti-inflammatory cannabimimetic effects *in vivo* (21). Hence, selective activation of CB2 in immune cells may be a strategy to reduce inflammatory responses.

Recently, we have demonstrated that *in vitro* incubation with BCP to PBMC of dairy cows affected the inflammatory response following a lipopolysaccharide (LPS)-challenge: BCP inhibited gene expression of nuclear factor kappa B subunit 1 (NFκB1), TNFα, suppressed IL-10, and tended to reduce cyclic-adenosine monophosphate (cAMP) in medium, possibly indicating CB2 activation (22). Moreover, proteomic analysis of LPS-activated monocyte-enriched cells revealed immune-related proteins and canonical pathways enriched by BCP, together indicative of immune-modulating effects of BCP in leukocytes from dairy cows (22). Thus, supplementation of BCP to activate CB2 may be a novel strategy to reduce inflammation and improve health of dairy cattle.

In the present study, we hypothesized that dietary supplementation with BCP may modulate the immune function by CB2-mediated responses in leukocytes, toward an anti-inflammatory response in heat-stressed cows. We further hypothesized that this effect may be enhanced synergistically by increased CS frequency, with potential implications for animal welfare and stress responses under environmental heat load.

## 2 Materials and methods

### 2.1 Animals and experimental procedures

The experimental protocol for this study was approved by the Volcani Center Animal Care Committee (approval number 1015/24 IL). The experiment was conducted for 5 weeks during peak summer (August-September) at the individual-feeding barn of the Volcani Institute dairy farm. Ambient temperature and relative humidity for THI calculations were recorded continually every 10 min within the experimental barn by a system that included 2 sensors installed at both sides of the barn (smaXtec Base station and climate sensors, Smart Farm Data, Hamilton, NZ). The average THI inside the barn during the experiment was 79.9 ± 0.052, which indicates severe heat load (1). The cows were group-housed in a covered pen with adjacent yard and fed *ad libitum* with a standard Israeli diet containing 16.5% crude protein and 1.78 Mcal of net energy for lactation (NE_L_) per kg of dry matter (DM).

Forty mid-lactation Holstein dairy cows were assigned according to their milk production, body weight, lactation number and days in lactation to one of 4 treatments: cows were divided randomly into controls (CTL, *n* = 20) or supplemented with BCP (*n* = 20, at 5 mg/kg BW), and half of the cows from each dietary treatment were cooled with either 3 or 6 cooling sessions (CS) a day, creating a 2 × 2 factorial design (*n* = 10 in each group): (1) CTL-6CS: control diet, 6 cooling sessions/day, (2) CTL-3CS: control diet, 3 cooling sessions/day, (3) BCP-6CS: control diet plus BCP supplementation at 5 mg/kg BW (dose based on (21)), 6 cooling sessions/day, and (4) BCP-3CS: control diet plus BCP supplementation at 5 mg/kg BW, 3 cooling sessions/day. Both CS frequency and BCP supplementation continued throughout the 5-week period. BCP was supplemented by adding it directly onto the individual feed of each cow right before cows were tied for feeding; BCP amount was adjusted weekly for each cow according to her average BW. Cooling sessions consisted of repeated cycles of water showers for 30 sec followed by 4.5 min forced ventilation (total of 45 min/session). 3CS cows were cooled before milkings at 04:45, 12:45 and 19:30 h; while 6CS cows were cooled at 04:30, 11:00, 12:30, 15:30, 19:15 and 22:00 h.

The cows were equipped with collar-mounted tags (HR-Tags; SCR Engineers Ltd., Hadarim, Netanya, Israel) that monitored rumination time, and with Pedometer Plus sensors (S.A.E. Afikim) that monitored lying time. The cows were milked three times a day beginning at 05:00, 14:00 and 20:30 h, and weighed automatically after each milking (S.A.E. Afikim), and milk production and BW data were collected daily. Heat stress indicators (rectal temperature, and respiration rate) were measured twice a week at 10:30 h throughout the experiment period. Milk samples were analyzed for milk composition and SCC (standard IDF 141C:2000; IDF, 2000) twice during the experiment.

### 2.2 Intra-mammary LPS challenge

At week 5 of the experiment, a subset of six cows from each group (24 cows total) were randomly assigned to an intramammary LPS challenge as follows: each cow was administered with a sterile preparation of 100 µg LPS from *E. coli* O127:B8 (Cat. No. L4516-1MG; Sigma-Aldrich, St. Louis, Missouri, USA) in 5 mL phosphate-buffered saline (PBS) via intramammary infusion. For each cow, one quarter of the udder was selected based on preceding SCC analysis from a milk sampling that was conducted at week 4 of the study prior to the challenge, in which we measured SCC in each quarter, to validate that the selected quarter had low SCC values. LPS administration was performed in the milking parlor immediately after the morning milking session at 05:15 h for the 6CS group and at 05:45 h for the 3CS group. The selected teat was cleaned with 70% alcohol and wiped. Then, an 18G blunt catheter was inserted into the teat orifice to administer the LPS solution. Afterward, the teat orifice was occluded, and the quarter was gently massaged to distribute the LPS solution.

Vaginal temperatures were measured continuously (every 10 min) for a week starting at 24 h pre-LPS using iButton temperature loggers (Cat. No. DS1922L; Maxim Integrated, San Jose, California, USA). Mean vaginal temperatures against time (min) were plotted for each treatment and area under the curve (AUC) was calculated using the trapezoidal rule: The area between two subsequent time-points was calculated using the trapezoid area formula ( $A=\frac{a+b}{2}h$), and the total AUC is the sum of the trapezoid areas.

Blood samples were collected from the coccygeal vein into Vacutainer tubes containing lithium heparin at 0.5, 5, 7.5, 10.25, 14.5, 24 h after LPS-administration; plasma was separated by centrifugation at 3000 ×*g* at 4°C for 10 min and stored at -80°C for analysis. Plasma concentrations of cortisol, glucose, haptoglobin and malondialdehyde (MDA), cytokines and chemokines and D2Dx immune test were analyzed at time points up to 24 h post-LPS. Additional blood samples were collected pre- (at week 4) and post-LPS at indicated times for cytokines and chemokines and for flow cytometry analysis of white blood cell (WBC) sub-populations. A diagram describing the experimental procedures is presented in **Figure 1**.

**Figure 1:**
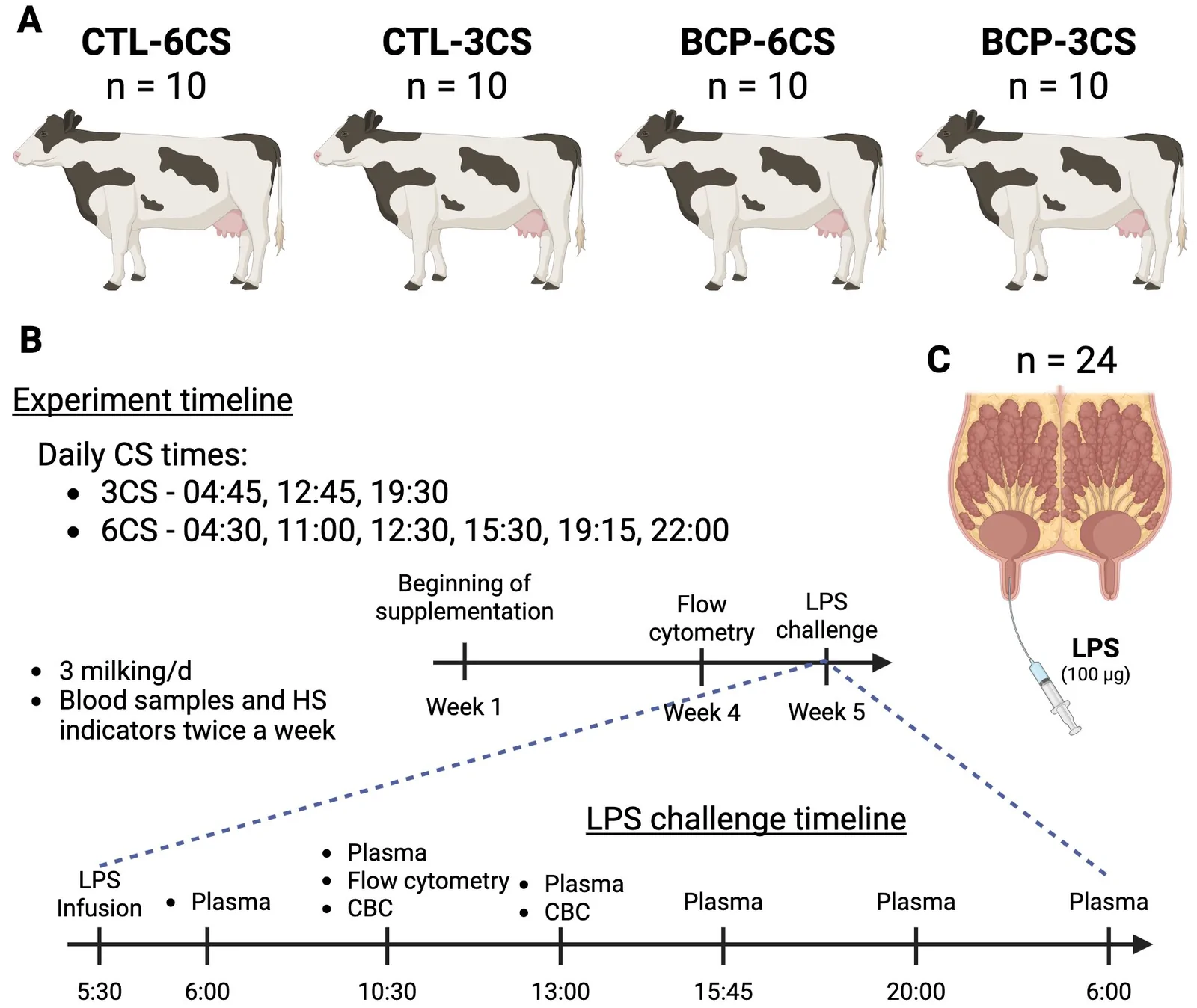
Diagram of experimental procedures: **(A)** forty mid-lactation Holstein dairy cows exposed to peak summer conditions were assigned according to their milk production, body weight, lactation number and days in lactation to one of 4 treatments: cows were divided into either controls (CTL, *n* = 20) or supplemented with BCP (BCP, *n* = 20, at 5 mg/kg BW), and half of the cows from each treatment were cooled with either 3 or 6 cooling sessions (CS) a day, creating a 2 × 2 factorial design (*n* = 10 in each group): CTL-6CS, CTL-3CS, BCP-6CS, and BCP-3CS. **(B)** Blood samples were taken and heat stress indices were measured twice a week for 4 weeks of the study. **(B, C)** At week 5, 24 cows (*n* = 6 from each group) were administered with LPS (100 µg LPS from *E. coli* in 5 ml PBS) by infusion into one quarter of the udder. Blood samples were taken at the indicated timepoints for analysis of different parameters. The figure was prepared using https://BioRender.com/85u21pr.

### 2.3 Inflammatory and oxidative stress markers in plasma

Cortisol ELISA kit (Cat. No. EIA-1887; DRG, Springfield, New Jersey, USA) and Bovine haptoglobin ELISA kit (Cat. No. E-10HPT; ICL Inc., Portland, OR, USA) were used to determine concentrations of these compounds in plasma. The sensitivity of the cortisol assay was 1.3 ng/mL, and the intra- and inter-assay coefficients of variation for the cortisol assay were 6.7 and 8.1%, respectively. Cortisol AUC was calculated by the trapezoidal rule as explained above. Glucose concentrations were determined using Cobas c111 analyzer (Roche diagnostics, Basel, Switzerland) with the reagent set GLUC2 (REF: 04657527190), using two enzymatic reactions with hexokinase and glucose-6-phosphate dehydrogenase to generate a UV emission that is correlated with the glucose concentration in the sample; results were calibrated against known glucose concentrations. MDA was measured by ThioBarbituric Acid Reactive Substance assay, the intra- and inter-assay for MDA were 9.4% and 2.5%, respectively. D2Dx immunity test, evaluating systemic immune function, was performed using a commercial kit (Cat. No. D2Dx-kt-100; Nano Discovery Inc., Orlando, FL, USA) and a CT-100 reader (Nano Discovery Inc., Orlando, FL, USA) according to the manufacturer’s protocol.

### 2.4 Cytokines and chemokines concentrations

Plasma concentrations of 10 bovine cytokines and chemokines [Interferon γ (IFNγ), IL-1α and β, IL-6, IL-10, macrophage inflammatory protein (MIP) -1α and -1β, interferon γ-induced protein 10 (IP-10), monocyte chemoattractant protein-1 (MCP-1), and TNFα] were determined using a commercial bovine cytokine/chemokine bead-based multiplex assay (Cat. No. BCYT1-33K; Sigma-Aldrich, St. Louis, Missouri, USA), which was validated by (23).

### 2.5 Hematology analysis

The CBC was analyzed at the hematology laboratory of the Veterinary Hospital of the Hebrew University (Rishon LeZion, Israel) to evaluate amounts and percentages of WBC, red blood cells (RBC) and other blood components.

### 2.6 Flow cytometry analysis of peripheral WBC populations

Changes in subpopulations of immune cells following LPS-challenge were evaluated by flow cytometry using the following antibody-fluorophore conjugates: CD4:Alexa Fluor 647 (A647) (Cat. No. MCA1653A647; Bio-Rad) – marker for T helper cells (24); CD8:R-phycoerythrin (RPE) (Cat. No. MCA837PE; Bio-Rad) – marker for cytotoxic T-cells (25); CD14:Fluorescein Isothiocyanate (FITC) (Cat. No. MCA2678F; Bio-Rad) – marker for classical monocytes (26, 27); CD16:A647 (Cat. No. MCA5665A647; Bio-Rad) – marker for non-classical monocytes and natural killer (NK) cells (28); CD335:A647 (Cat. No. MCA2365A647; Bio-Rad) – marker for NK cells (29); WC1:FITC (Cat. No. MCA1655F; Bio-Rad) – marker for γδ T-cells (30). From each cow, 6 mL of blood were taken from jugular vein using a heparin coated tube at week 4 (pre-LPS) and at 5 h post-LPS and processed immediately as follows: Blood was centrifuged at 1400×*g* at 4°C for 8 min to remove plasma layer. RBC were then lysed by adding 9 mL double distilled water (DDW) for 1 min. The lysis was stopped by adding 1 mL 10×PBS. The WBCs were concentrated by centrifugation at 400×*g* at 4°C for 10 min and resuspended in 1 mL of 1×PBS. Their viability and total number in each sample was assessed using an automatic cell counter (Countess III; Thermo Fisher) and then their concentrations adjusted to 8×10^5^ live cells/mL. For each cow, 100 µL of the WBC cell suspension was incubated for 30 min at 4°C with the following 2-color antibody preparations (7 µL of each antibody): 1) CD4:A647 + CD8:RPE; 2) CD14:FITC + CD16:A647; 3) CD335:A647 + WC1:FITC; 4) Control (unstained). Following the incubation, the cells were washed with 3 mL cold 1×PBS, centrifuged at 400×*g* at 4°C for 7 min, and resuspended in 300 μL of 1×PBS. The samples were then analyzed using flow cytometer machine (BD Accuri c6 plus), which was set to count 10,000 events for each sample. For antibody pairing, the fluorophore combinations were selected to prevent spectral overlap, thus no compensation was required: A647 fluorophore was excited by the 640 nm laser and emission was detected through the 670 LP filter, while FITC and RPE were excited by the 488 nm laser, and their emission was detected through 533/30 and 585/40 filters, respectively. Gating thresholds were determined by using unstained samples as internal controls to account for individual cellular autofluorescence. The results were analyzed using FlowJo software (Version 10; BD Life Sciences). For each cell subpopulation we calculated the percentage of positive reads from the total cells and analyzed for differences between the treatment groups. An example of the gating strategy and representative dot plots are shown in **Supplementary Materials** (**Supplementary Figure 1**).

### 2.7 Statistical analysis

Continuous variables (dry matter intake, eating time, rumination time, respiratory rate, panting time, resting time, resting bout duration, rectal temperature, milk production, SCC, milk components, vaginal temperatures, plasma concentrations of cortisol, glucose, haptoglobin, MDA, D2Dx score and plasma concentrations of cytokines and chemokines) were analyzed as repeated measurements by PROC MIXED model in SAS (version 9.2; SAS Institute Inc., Cary, NC, USA). The model was specified as:


$$Y_{ijlm}=µ+B_{j}+H_{l}+T_{m}+B_{j}H_{l}+B_{j}T_{m}+H_{l}T_{m}+B_{j}H_{l}T_{m}+C_{i(jl)}+E_{ijlm}$$

where Y_ijlm_ = dependent variable; µ = overall mean; B_j_ = fixed effect of BCP; H_l_ = fixed effect of CS; T_m_ = fixed effect of time; B_j_H_l_ = fixed effect of the interaction between BCP and CS; B_j_T_m_ = fixed effect of the interaction between BCP and time; H_l_T_m_ = fixed effect of the interaction between CS and time; B_j_H_l_T_m_ = fixed effect of the interaction between BCP, CS and time, and C_i(jl)_ = random effect of cow nested within treatment; E_ijlm_ = residual error. The main effects of BCP, CS, time, and their interactions were analyzed. In analyses of cytokines and chemokines, the basal values in time 0 were used as a covariance in the model. The model assumptions of normality of variances were tested by the Shapiro-Wilk test on the model residuals.

Data as specific time points (flow cytometry and CBC data, vaginal temperature AUC and cortisol AUC) were analyzed as repeated measurements using the General Linear Models procedure of SAS (version 9.2, SAS Institute, Cary, NC, 2002). The model included the effect of dietary treatment (CTL and BCP), cooling frequency (3CS and 6CS), the random effect of cow nested within treatment, and the interaction between BCP and CS. In flow cytometry data, we also analyzed the effect of LPS.

Autoregressive order 1 (AR 1) was used as a covariance structure in the model because it resulted in the lowest Bayesian information criterion for most of the tested variables. Significance is declared at *P* ≤ 0.05 with a tendency noted at 0.05 < *P* ≤ 0.10 by Bonferroni test.

## 3 Results

### 3.1 Bioavailability of oral BCP and effects of BCP and CS frequency on production and behavior indices

Oral supplementation of BCP at 5 mg/kg BW increased plasma BCP concentrations compared to CTL at week 4 of the study (1.82 vs. 0.00 ppb in BCP-supplemented vs. controls, respectively, SEM = 0.316; *P* < 0.0001). As shown in **Table 1**, BCP supplementation for 4 weeks (pre- LPS-challenge) did not affect dry matter intake (DMI), milk yield, milk composition, panting time and rectal temperature, whereas rumination time (*P*_BCP_ = 0.06) and respiratory rate (*P*_BCP_ = 0.10) tended to be affected by BCP (**Table 1**). BCP reduced eating time (149.7 vs. 186.9 min/d in BCP and CTL, respectively, SEM = 10.71, *P*_BCP_ = 0.01) in BCP-supplemented cows compared to controls (**Table 1**). Moreover, BCP-supplemented cows had a longer duration of resting bouts compared to CTL (*P*_BCP_ = 0.005) with no effect of cooling frequency (*P*_CS_ = 0.62), while the interaction of BCP×CS was significant (*P_BCP×CS_* < 0.0001; **Table 1**).

**Table 1:** Effects of BCP and cooling frequency on feeding behavior, welfare indices and milk production over 4 weeks of the study.

Parameters	Treatment groups				SEM	Main effect *p*-value						
	CTL-6CS (*n* = 10)	CTL-3CS (*n* = 10)	BCP-6CS (*n* = 10)	BCP-3CS (*n* = 10)		BCP	CS	BCP×CS	Time	Time×BCP	Time×CS	Time×BCP×CS
DMI (kg/d)	26.1	25.1	26.4	25.5	0.48	0.40	0.81	0.75	0.05	NS	NS	NS
Eating time (min/d)	187.7	186.1	158.1	141.3	14.77	0.01	0.53	0.60	NS	NS	NS	NS
Rumination time (min/d)	510.6^a^	454.4^b^	520.8^a^	425.1^b^	8.91	0.06	<0.0001	0.40	0.05	NS	NS	NS
Respiratory rate (counts/min)	64.7^b^	84.9^a^	62.8^b^	87.5^a^	2.71	0.10	0.004	0.96	NS	0.05	NS	NS
Panting time (min/d)	266.1^ab^	350.1^a^	187.2^b^	371.7^ab^	39.64	0.45	0.001	0.20	0.03	NS	NS	NS
Rest time (min/d)	551.1^a^	500.2^b^	536.2^ab^	552.7^a^	15.33	0.69	0.05	0.02	NS	NS	NS	NS
Resting bouts duration (min/bout)	48.9 ^a^	44.2^b^	47.0^ab^	52.9^a^	1.40	0.005	0.62	<0.0001	NS	NS	NS	NS
Rectal temperature (°C)	38.9	38.5	38.4	38.6	0.17	0.25	0.14	0.17	NS	NS	NS	NS
Milk (kg/d)	42.23^a^	40.53^b^	43.63^a^	40.23^b^	0.46	0.15	0.56	0.03	0.02	NS	<0.0001	NS
LogSCC	5.8^b^	8.0^a^	7.0^ab^	6.4^ab^	0.48	0.27	0.05	0.18	NS	NS	NS	NS
Lactose (%)	4.9^a^	4.7^b^	4.8^ab^	4.7^b^	0.05	0.72	0.01	0.93	0.0002	NS	NS	NS
Protein (%)	3.3	3.1	3.2	3.3	0.08	0.26	0.12	0.57	0.0009	NS	0.04	NS
Fat (%)	3.9	3.8	3.7	3.8	0.22	0.17	0.08	0.65	<0.0001	NS	NS	NS

Forty mid lactation cows were randomly allocated into four treatment groups: (1) CTL-6CS (control diet, 6 cooling sessions/d), (2) CTL-3CS (control diet, 3 cooling sessions/d), (3) BCP-6CS (BCP supplemented at 5 mg/kg BW, 6 cooling sessions/d), and (4) BCP-3CS (BCP supplemented at 5 mg/kg BW, 3 cooling sessions/d). Physiological parameters were measured twice a week per cow throughout weeks 1 to 4 of treatment. Means with different superscript letters differ significantly (*P* ≤ 0.05). SEM, Standard error of mean; BCP, β-Caryophyllene supplemented diet; CS, Cooling sessions frequency; CTL, Control; DMI, Dry matter intake; SCC, Somatic cell count; NS, non-significant; Time, days in experiment; all interactions with time that were not significant were removed from the statistical model.

When comparing cooling regimens across dietary treatments, cows that were in 6CS had higher milk lactose concentration (4.83 vs. 4.69% in 6CS and 3CS, respectively, SEM = 0.032, *P*_CS_ = 0.01; **Table 1**). In addition, rumination time was longer in 6CS than in 3CS (515.7 vs. 439.7 min/d in 6CS and 3CS, respectively, SEM = 6.49, *P*_CS_ < 0.0001; **Table 1**). On the other hand, 6CS decreased panting time (226.7 vs. 360.9 min/d, in 6CS and 3CS, respectively, SEM = 28.91, *P*_CS_ = 0.001; **Table 1**) and respiratory rate (63.8 vs. 86.2 counts/min, in 6CS and 3CS, respectively, SEM = 2.16, *P*_CS_ = 0.004; **Table 1**), all indicators for heat stress. In addition, cows in 6CS had longer resting time (*P*_CS_ = 0.05) and lower LogSCC (*P*_CS_ = 0.05) than cows in 3CS (**Table 1**). The BCP × CS interaction was not significant for most of the production and welfare parameters, except for resting time (*P*_BCP×CS_ = 0.02), resting bouts duration (*P*_BCP×CS_ < 0.0001) and milk yield (*P*_BCP×CS_ = 0.03; **Table 1**). The effect of time (days in study) was significant for DMI, rumination time, panting time, milk production and milk components, while most of the interactions of time with dietary treatment or CS were not significant, except for respiratory rate for BCP×time (*P*_Time×BCP_ = 0.05), and milk yield (*P*_Time×CS_ < 0.0001) and milk protein for CS×time (*P*_Time×CS_ = 0.04; **Table 1**).

### 3.2 Effects of BCP and CS frequency on vaginal temperature, stress and inflammatory markers in blood during LPS-challenge

The average vaginal temperatures of the cows at 2 h before the LPS-challenge were similar among groups (*P* > 0.6). Following LPS-administration, cows’ vaginal temperature rapidly increased, reaching peak fever between 4.5 and 7 hours post LPS-infusion, and returning to near basal levels after approximately 10 hours. Interestingly, cows supplemented with BCP had 0.45°C lower vaginal temperatures compared to the controls around the timing of peak fever, with lowest temperatures in the BCP-6CS group (*P*_BCP_ < 0.05; **Figure 2A**). Moreover, the AUC of vaginal temperatures was reduced in the BCP groups compared to CTL (1981.1 vs. 1998.2 °C × min, respectively; SEM = 5.97; *P*_BCP_ = 0.01; **Figure 2B**). There was no effect of CS (*P*_CS_ = 0.30) or BCP × CS interaction on vaginal temperature AUC (*P*_BCP×CS_ = 0.44). Cows with 6CS had lower vaginal temperature compared to 3CS cows at the time of peak fever (*P*_CS_ < 0.05; **Figure 2A**).

**Figure 2:**
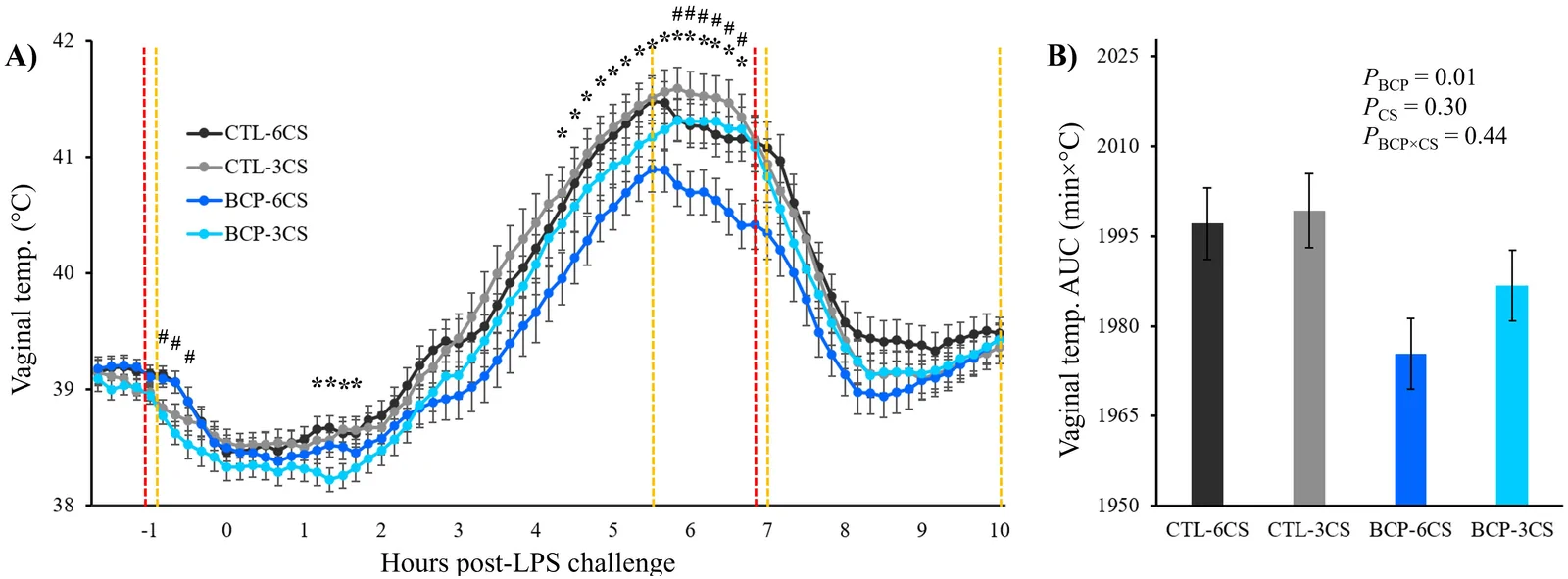
Vaginal temperatures following an intra-mammary LPS challenge. **(A)** Time-course changes in vaginal temperatures measured at 10 min intervals from 1:40 hours pre-infusion to 10 h post-infusion. Twenty-four mid lactation cows were allocated into four treatment groups: (1) CTL-6CS (control diet, 6 cooling sessions/d), (2) CTL-3CS (control diet, 3 cooling sessions/d), (3) BCP-6CS (BCP supplemented at 5 mg/kg BW, 6 cooling sessions/d), and (4) BCP-3CS (BCP supplemented at 5 mg/kg BW, 3 cooling sessions/d). At week 5, six cows from each group were administered with LPS (100 µg LPS from *E. coli* in 5 ml PBS) by infusion into one quarter of the udder. Vaginal temperatures were monitored continuously using iButton temperature loggers. Dashed lines indicate cooling sessions – 6CS (Orange); 3CS (Red). Asterisk (*) highlights timepoints where vaginal temperature in BCP-treated cows was significantly different from CTL animals (*P*_BCP_ ≤ 0.05); (#) highlights timepoints where vaginal temperature in cows receiving 6 cooling sessions was significantly different from that of cows receiving 3 cooling sessions (*P*_CS_ ≤ 0.05). **(B)** Cumulative vaginal thermal responses to LPS exposure, calculated as area under the curve (AUC) over the time presented in **(A)** Data represent the mean ± SEM. CTL, Control diet; BCP, β-Caryophyllene supplemented diet; CS, Cooling sessions per day (3 or 6); LPS, lipopolysaccharide.

During the LPS-challenge, the main effects of BCP, CS and their interactions on average cortisol concentrations in plasma were not significant (**Table 2**); although BCP-6CS cows had numerically the lowest concentrations of cortisol compared to the other groups (**Figure 3A**). At 5 h post-LPS cortisol concentrations tended to be lower in BCP-6CS than in CTL-3CS (76.8 vs. 148.5 ng/ml, respectively, SEM = 25.42, *P* = 0.06), as well as at 14.5 h post-LPS (12.4 vs. 46.7 ng/ml, in BCP-6CS and in CTL-3CS respectively, SEM = 13.04, *P* = 0.07), and at 14.5 and 24 h post-LPS, cortisol concentrations were lower in BCP-6CS than in CTL-3CS (8.2 vs. 35.7 ng/ml, SEM = 7.66, *P* = 0.01). In addition, the AUC of cortisol levels tended to be lower in BCP-6CS than in CTL-3CS (551.1 vs. 1333.3 ng/ml × h in BCP-6CS and CTL-3CS, respectively, SEM = 288.39, *P* = 0.07; **Figure 3B**).

**Table 2:** Effects of BCP and cooling frequency on levels of stress and inflammation markers in blood plasma following LPS administration.

Parameters	Treatment groups				SEM	Main effect *p*-value			
	CTL-6CS (*n* = 6)	CTL-3CS (*n* = 6)	BCP-6CS (*n* = 6)	BCP-3CS (*n* = 6)		BCP	CS	BCP×CS	Time
Cortisol (ng/mL)	27.72	54.30	25.55	37.61	8.505	0.54	0.14	0.72	0.003
Glucose (mg/dL)	69.21	70.18	68.82	70.70	1.805	0.99	0.79	0.19	0.19
Haptoglobin (mg/dL)	29.31	28.71	30.32	31.25	7.208	0.93	0.92	0.89	<0.0001
MDA (µM)	1.35	1.67	1.81	1.90	0.293	0.23	0.72	0.31	0.0002
D2Dx Score (AU)	0.06	0.07	0.06	0.06	0.005	0.27	0.60	0.98	<0.0001
TNFα (pg/mL)	1214.67	966.11	1129.71	1082.09	230.703	0.99	0.42	0.55	0.26
IL-6 (pg/mL)	1488.30	1196.42	1151.88	1233.94	290.715	0.62	0.72	0.53	0.0003
IL-10 (pg/mL)	289.50	313.14	238.88	244.35	55.923	0.31	0.98	0.89	0.004
IFNγ (pg/mL)	0.87	0.81	0.81	0.85	0.142	0.92	0.81	0.53	0.002
IL-1α (pg/mL)	7.15	6.60	7.64	8.55	1.676	0.33	0.33	0.34	0.006
IL-1β (pg/mL)	10.98	8.47	11.80	11.14	2.565	0.24	0.76	0.64	0.81
MIP-1α (pg/mL)	62.66	59.06	64.55	71.86	5.930	0.21	0.67	0.20	0.01
MIP-1β (pg/mL)	259.68	569.31	172.24	324.46	129.635	0.21	0.05	0.46	0.005
IP-10 (pg/mL)	1061.52	794.10	711.57	849.67	110.048	0.12	0.46	0.04	<0.0001
MCP-1 (pg/mL)	1304.22	1300.25	1006.95	1217.78	195.255	0.35	0.70	0.49	0.0001

Blood samples were collected at 0.5, 5, 7.5, 10.25, 14.5 and 24 hours after the intra-mammary LPS administration for the analysis of cortisol, glucose, haptoglobin, MDA and D2Dx. Plasma cytokine/chemokine analysis on samples collected at 0.5, 5, 7.5, 14.5 and 24 hours post-LPS. Values represent mean concentrations across of all the time-points. Means with different superscript letters differ significantly (*P* ≤ 0.05). SEM, Standard error of mean. CTL, Control diet; BCP, β-Caryophyllene supplemented diet; CS, Cooling sessions per day (3 or 6); LPS, Lipopolysaccharide; AU, Arbitrary units; D2Dx Score, D2Dx™ immunity test (Nano Discovery Inc., Orlando, FL, USA); IFNγ, Interferon gamma; IL, Interleukin; IP-10, Interferon gamma-induced protein 10; MCP-1, Monocyte Chemoattractant Protein-1; MDA, Malondialdehyde; MIP-1α, Macrophage Inflammatory Protein-1 alpha; MIP-1β, Macrophage Inflammatory Protein-1 beta; TNFα, Tumor necrosis factor alpha. Time, hours post-LPS; All the interactions of time with BCP, CS and BCP×CS were not significant for these parameters.

**Figure 3:**
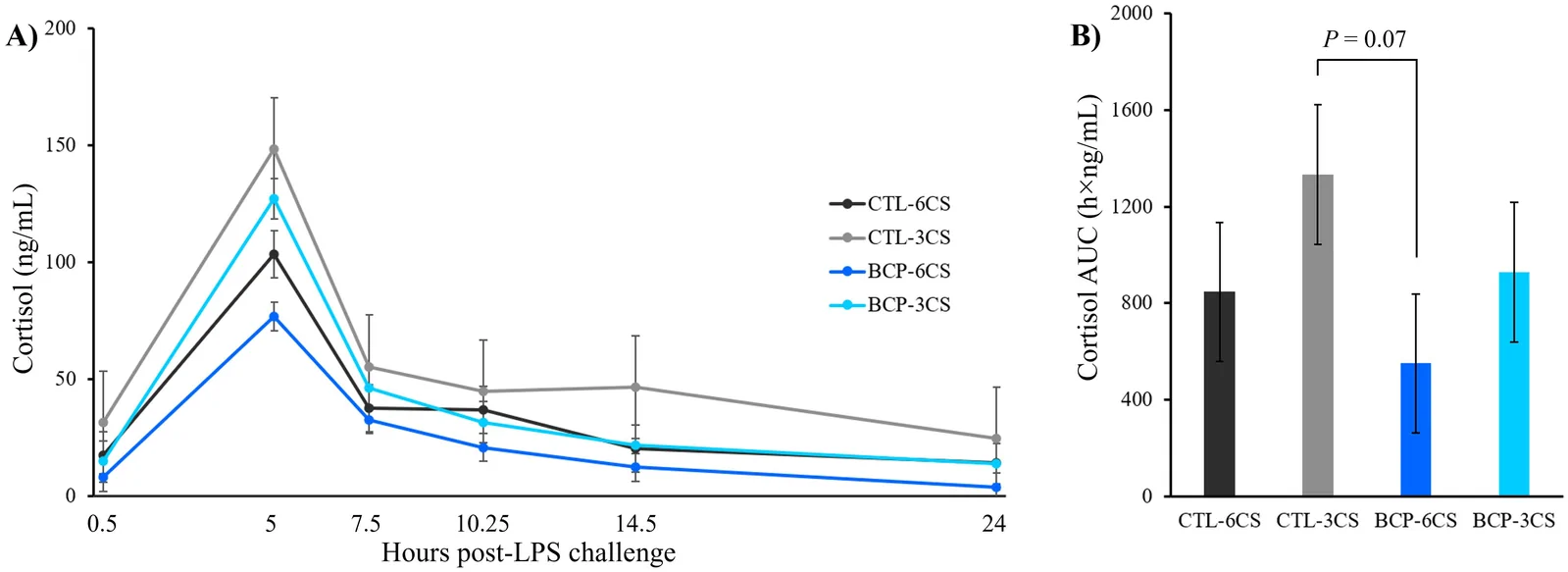
Changes in plasma cortisol following acute exposure to LPS. **(A)** Blood samples were collected from cows of the four treatment groups (*n* = 6 per group) at 0.5, 5, 7.5, 10.25, 14.5 and 24 hours after the intra-mammary LPS administration and plasma cortisol levels were analyzed. **(B)** Cumulative cortisol concentrations post-LPS exposure, calculated as area under the curve (AUC) over the time presented in **(A)**. CTL = Control diet; BCP = β-Caryophyllene supplemented diet; CS = Cooling sessions per day (3 or 6); LPS = Lipopolysaccharide.

Plasma glucose and haptoglobin concentrations, MDA and D2Dx values were not different among treatments (**Table 2**). When analyzing plasma concentrations of cytokines and chemokines, we observed a reduction in MIP-1β concentrations in the BCP cows compared to CTL at 14.5 and 24 hours after LPS (22.6 vs. 31.3 pg/mL in BCP and CTL, respectively, SEM = 2.80, *P*_BCP_ = 0.05). The basal concentrations of cytokines and chemokines pre-LPS were similar among groups, and the effects of BCP on cytokines and chemokines concentrations post-LPS were not statistically significant (IFNγ, IL-1α, IL-1β, IL-6, IL-10, MIP-1α, IP-10, MCP-1, TNFα; **Table 2**; **Figure 4**).

**Figure 4:**
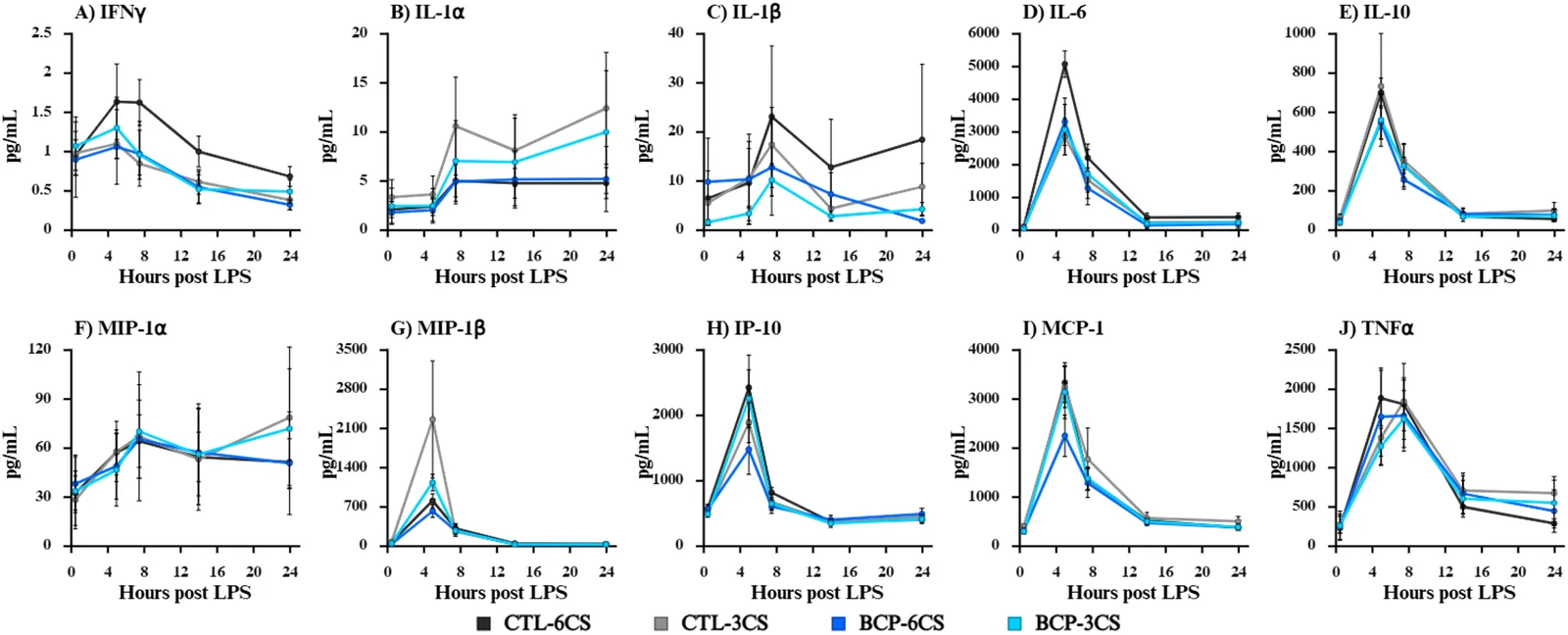
Changes in plasma concentrations of cytokines and chemokines following acute exposure to LPS. **(A)** IFNγ; **(B)** IL-1α; **(C)** IL-1β; **(D)** IL-6; **(E)** IL-10; **(F)** MIP-1α; **(G)** MIP-1β; **(H)** IP-10; **(I)** MCP-1; **(J)** TNFα. Blood samples were collected from cows of the four treatment groups (*n* = 6) at 0.5, 5, 7.5, 14.5 and 24 hours after the intra-mammary LPS administration and analyzed for plasma cytokines and chemokines concentrations using MILLIPLEX® Bovine Cytokine/Chemokine Magnetic Bead Panel (Merck Millipore). Data represent the mean ± SEM. CTL, Control diet; BCP, β-Caryophyllene supplemented diet; CS, Cooling sessions per day (3 or 6); LPS, Lipopolysaccharide; IFNγ, Interferon gamma; IL, Interleukin; IP-10, Interferon gamma-induced protein 10; MCP-1, Monocyte Chemoattractant Protein-1; MIP-1α, Macrophage Inflammatory Protein-1 alpha; MIP-1β, Macrophage Inflammatory Protein-1 beta; TNFα, Tumor necrosis factor alpha. The figure was prepared using https://BioRender.com/b7hkcx8.

Plasma concentrations of cortisol, glucose, haptoglobin, MDA, D2Dx values, and cytokines and chemokines were not affected by cooling frequency, except for MIP-1β which was lower in 6CS compared to 3CS (214.6 vs. 447.4 pg/mL in 6CS and 3CS, respectively, SEM = 91.13, *P*_CS_ = 0.05; **Table 2**). The interactions of BCP × CS were not significant for these parameters, except for IP-10 (*P*_BCP×CS_ = 0.04; **Table 2**). The effect of time (hour post-LPS) was significant for cortisol, haptoglobin, MDA, D2Dx score, IL6, IFNγ, IL1α, IL10, MIP1α, MIP1-β, IP-10 and MCP-1 (**Table 2**), however all the interactions of time with dietary treatment or CS were not significant for these parameters.

### 3.3 Effects of LPS, BCP and cooling frequency on populations of blood and immune cells during the LPS-challenge

When comparing immune cell populations pre- vs. post-LPS challenge by flow cytometry, we found the percentages of CD4+ in blood increased at 5 h post-LPS compared to pre-LPS (*P*_LPS_ = 0.023), as well as decreased populations of CD14+, CD16+, and CD14+CD16+ in blood (*P*_LPS_ < 0.0001; **Figure 5**). Pre-LPS, BCP × CS interactions were significant for CD335+ (*P*_BCP×CS_ = 0.02) and for CD335+WC1+ (*P*_BCP×CS_ = 0.01) cells, with a tendency of an interaction for WC1+ (*P*_BCP×CS_ = 0.06). Post-LPS, BCP × CS interactions were significant for CD16+ (*P*_BCP×CS_ = 0.003), CD335+ (*P*_BCP×CS_ = 0.05), WC1+ (*P*_BCP×CS_ = 0.03), and CD335+WC1+ cells (*P*_BCP×CS_ = 0.03), with tendencies for interactions for CD4+CD8+ (*P*_BCP×CS_ = 0.07), CD14+ (*P*_BCP×CS_ = 0.10), CD4+ (*P*_BCP×CS_ = 0.10), and CD14+CD16+ (*P*_BCP×CS_ = 0.09), indicating that the combination of BCP and CS reshaped innate (CD14+, CD16+, and CD335+) and γδ-related (WC1+) lymphocyte responses to the LPS challenge. The basal percentages of immune cell populations pre-LPS were similar among all groups, and we did not detect significant main effects of BCP or CS on immune cell populations by flow cytometry (**Figure 5**).

**Figure 5:**
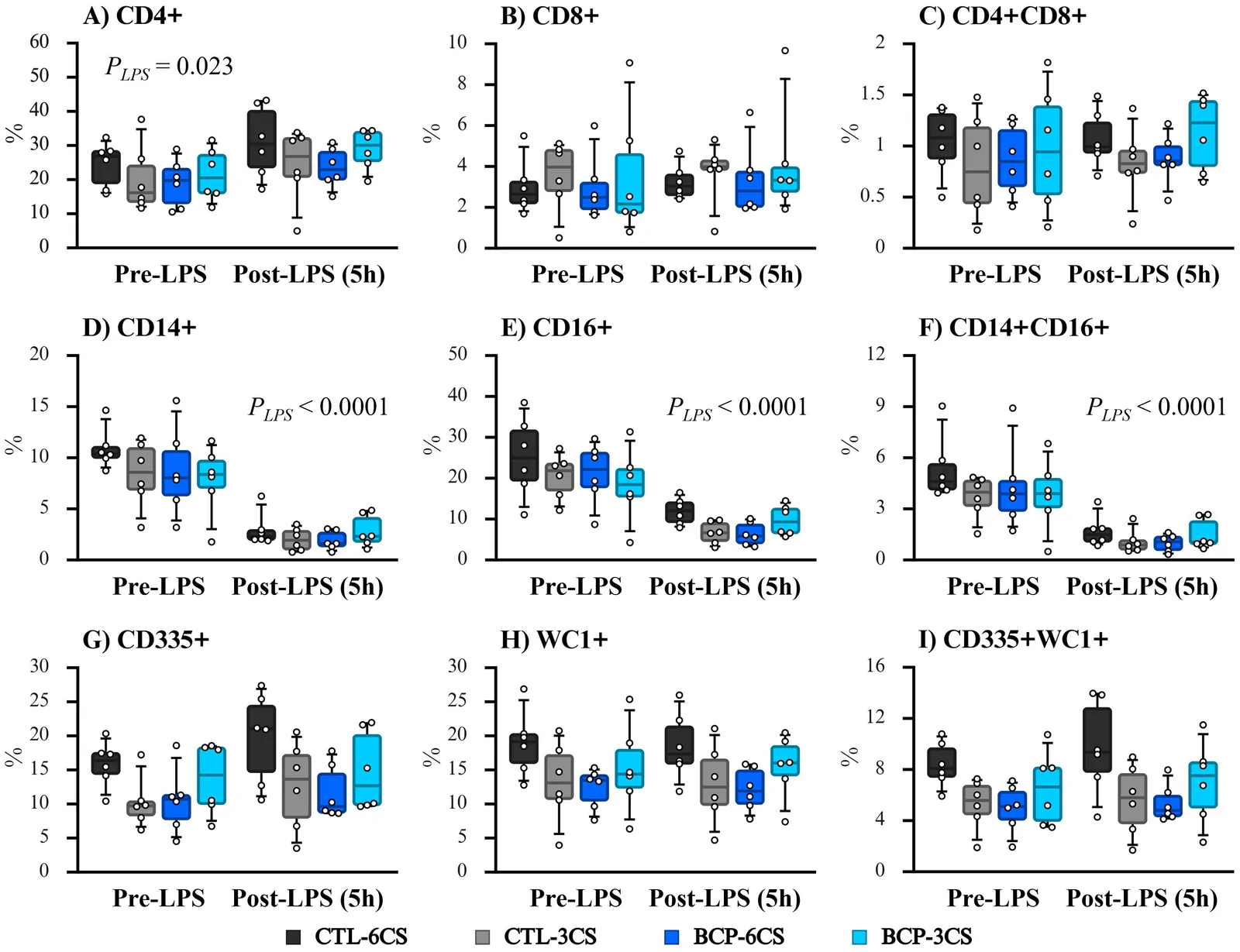
Changes in immune cells populations in blood following acute exposure to LPS. **(A)** CD4+; **(B)** CD8+; **(C)** CD4+CD8+; **(D)** CD14+; **(E)** CD16+; **(F)** CD14+CD16+; **(G)** CD335+; **(H)** WC1+; **(I)** CD335+WC1+. Blood samples were collected from cows of the four treatment groups (*n* = 6) at week 4 of the study and at 5 hours after the intra-mammary LPS administration and analyzed via Flow Cytometry for proportions of WBC subpopulations expressing indicated immune markers. Each sample was normalized and gated according to its own unstained controls. Data is presented as boxplots (whiskers show 5th–95th percentiles) with individual data points. CTL = Control diet; BCP = β-Caryophyllene supplemented diet; CS = Cooling sessions per day (3 or 6); LPS = Lipopolysaccharide; CD = cluster of differentiation; WC = workshop cluster. The figure was prepared using https://BioRender.com/aj0i0df.

Hematological analysis of complete blood counts indicated that at 5 h post-LPS, white blood cells, neutrophils and lymphocytes absolute counts and percentages did not differ between BCP or CS treatments, while monocyte percentages tended to be lower in BCP than in CTL (*P*_BCP_ = 0.06; **Table 3**). However, eosinophils (as a percentage of WBC) were increased for 6CS vs. 3CS (*P*_CS_ = 0.04), basophils counts were affected by a BCP × CS interaction (*P*_BCP×CS_ = 0.04), and basophil percentages tended to be affected by this interaction (*P*_BCP×CS_ = 0.06) and by BCP (*P*_BCP_ = 0.07; **Table 3**). At 7.5 h post-LPS, monocyte percentages were lower in BCP supplemented cows vs. CTL (1.79 vs. 2.80% in BCP and CTL respectively, SEM = 0.249, *P*_BCP_ = 0.02; **Table 3**), and the percentage of basophils tended to be higher in BCP than in CTL (*P*_BCP_ = 0.07). The amount and percentages of neutrophils tended to be higher in 6CS compared to 3CS (*P*_CS_ < 0.09; **Table 3**), with no effects of BCP or the interaction BCP × CS on these parameters (**Table 3**).

**Table 3:** Effects of BCP and cooling frequency on white blood cells count following LPS administration.

Parameter	Treatment groups				SEM	Main effect *p*-value		
	CTL-6CS (*n* = 6)	CTL-3CS (*n* = 6)	BCP-6CS (*n* = 6)	BCP-3CS (*n* = 6)		BCP	CS	BCP×CS
5 h post LPS								
White blood cells (10^3^/µL)	2.15	1.87	2.27	2.11	0.503	0.75	0.66	0.91
Neutrophils (10^3^/µL)	1.03	0.67	0.84	0.83	0.289	0.96	0.53	0.57
Monocytes (10^3^/µL)	0.04	0.03	0.02	0.05	0.015	0.93	0.61	0.36
Lymphocytes (10^3^/µL)	0.92	1.05	1.23	1.11	0.215	0.42	0.99	0.58
Eosinophils (10^3^/µL)	0.09	0.08	0.11	0.08	0.014	0.30	0.23	0.40
Basophils (10^3^/µL)	0.02	0.03	0.04	0.03	0.006	0.13	0.67	0.04
Large unstained cells (10^3^/µL)	0.01	0.01	0.01	0.02	0.006	0.09	0.61	0.61
Neutrophils (%)	40.50	37.70	36.06	37.38	4.796	0.64	0.87	0.68
Monocytes (%)	1.70	1.28	1.14	1.87	0.300	0.06	0.46	0.28
Lymphocytes (%)	50.48	53.73	54.82	54.02	4.681	0.64	0.79	0.68
Eosinophils (%)	6.05^a^	4.33^b^	5.54^a^	4.38^b^	0.650	0.68	0.04	0.68
Basophils (%)	1.03	1.67	1.88	1.65	0.210	0.07	0.31	0.06
Large unstained cells (%)	0.23	0.50	0.60	0.70	0.154	0.08	0.25	0.60
7.5 h post LPS								
White blood cells (10^3^/µL)	3.85	2.82	3.26	3.50	0.588	0.94	0.53	0.32
Neutrophils (10^3^/µL)	2.48	1.44	1.80	1.67	0.299	0.49	0.09	0.17
Monocytes (10^3^/µL)	0.09	0.08	0.06	0.06	0.018	0.28	0.75	0.75
Lymphocytes (10^3^/µL)	1.13	1.21	1.25	1.61	0.319	0.44	0.52	0.69
Eosinophils (10^3^/µL)	0.06	0.07	0.08	0.11	0.025	0.21	0.46	0.71
Basophils (10^3^/µL)	0.03	0.02	0.04	0.03	0.007	0.15	0.42	0.82
Large unstained cells (10^3^/µL)	0.04	0.01	0.02	0.02	0.009	0.55	0.20	0.29
Neutrophils (%)	64.63	52.45	57.30	40.35	7.183	0.22	0.08	0.76
Monocytes (%)	3.00^a^	2.60^a^	1.77^b^	1.80^b^	0.361	0.02	0.63	0.58
Lymphocytes (%)	29.23	41.65	36.73	43.35	5.545	0.44	0.13	0.62
Eosinophils (%)	1.45	2.08	2.37	2.95	0.508	0.12	0.28	0.97
Basophils (%)	0.73	0.65	1.10	0.93	0.157	0.07	0.46	0.76
Large unstained cells (%)	0.98	0.58	0.77	0.65	0.308	0.84	0.44	0.67

Blood samples were collected at 5 and 7.5 hours after the intra-mammary LPS administration. Cell count analysis was performed at the hematology laboratory of the Veterinary Hospital of the Hebrew University (Rishon LeZion, Israel). Means with different superscript letters differ significantly (*P* ≤ 0.05). SEM, Standard error of mean; CTL, Control diet; BCP, β-Caryophyllene supplemented diet; CS, Cooling sessions per day (3 or 6); LPS, Lipopolysaccharide.

Within the erythrogram and platelet panel of the CBC at 5 h post-LPS, significant BCP × CS interactions were detected for red blood cell count (*P*_BCP×CS_ = 0.04), hemoglobin distribution width (*P*_BCP×CS_ = 0.04), mean reticulocyte volume (*P*_BCP×CS_ = 0.05), reticulocyte cellular hemoglobin concentration mean (*P*_BCP×CS_ = 0.04), reticulocyte cellular hemoglobin (*P*_BCP×CS_ = 0.04), mean platelet mass (*P*_BCP×CS_ = 0.03), and platelet mass distribution width (*P*_BCP×CS_ = 0.02; **Table 4**). Moreover, BCP had significant effects on mean platelet mass (1.62 and 1.54 pg in BCP and CTL, respectively, SEM = 0.021, *P*_BCP_ = 0.02), on mean platelet volume (6.98 and 6.74 fL in BCP and CTL, respectively, SEM = 0.075, *P*_BCP_ = 0.003) and on platelet mass distribution width (0.62 and 0.55 fL in BCP and CTL, respectively, SEM = 0.018, *P*_BCP_ = 0.005; **Table 4**). At 7.5 h post-LPS, mean platelet volume was higher with BCP compared to CTL (7.19 and 6.93 fL in BCP and CTL, respectively, SEM = 0.079, *P*_BCP_ = 0.05; **Table 4**). At 5 h post-LPS, the CS frequency affected mean reticulocyte volume (*P*_CS_ = 0.04) and platelet distribution width (*P*_CS_ = 0.04; **Table 4**). Significant BCP × CS interactions were detected for RBC count at 7.5 h post-LPS (*P*_BCP×CS_ = 0.01), as well as for hemoglobin (*P*_BCP×CS_ = 0.03), and hematocrit (*P*_BCP×CS_ = 0.03; **Table 4**).

**Table 4:** Effects of BCP and cooling frequency on erythrogram and platelet profiles following LPS administration.

Parameter	Treatment groups				SEM	Main effect *p*-value		
	CTL-6CS	CTL-3CS	BCP-6CS	BCP-3CS		BCP	CS	BCP
	(*n* = 6)	(*n* = 6)	(*n* = 6)	(*n* = 6)				×CS
5 h post LPS								
Red Blood Cell count (10^6^/µL)	6.40^ab^	5.85^b^	6.12^ab^	6.94^a^	0.253	0.18	0.72	0.04
Hematocrit (%)	27.9	27.47	26.53	30.12	1.059	0.64	0.16	0.10
Mean Corpuscular Volume (fL)	43.68	47.07	43.43	43.62	1.196	0.15	0.17	0.13
Macrocyte (%)	0.9	2.17	1.45	1.05	0.399	0.50	0.33	0.14
Hemoglobin (g/dL)	10.07	10.07	9.64	10.82	0.39	0.64	0.92	0.16
Mean Corpuscular Hemoglobin (pg)	15.75	17.23	15.78	15.67	0.476	0.22	0.21	0.09
Mean Corpuscular Hemoglobin Concentration (g/dL)	36	36.6	36.37	35.9	0.294	0.63	0.82	0.33
Cellular Hemoglobin (pg)	15.05	16.4	15.12	15	0.463	0.18	0.22	0.13
Cellular Hemoglobin Concentration Mean (g/dL)	34.33	34.7	34.72	34.27	0.212	0.92	0.73	0.31
Hypochromic RBC (%)	1.35	0.9	0.88	1.25	0.271	0.80	0.86	0.47
Hyperchromic RBC (%)	0.83	0.65	0.82	0.88	0.087	0.34	0.40	0.28
Red Cell Distribution Width (%)	17.67	17.02	18.5	17.67	0.412	0.10	0.10	0.12
Hemoglobin Distribution Width (g/dL)	2.42^a^	2.29^b^	2.39^ab^	2.41^ab^	0.049	0.27	0.22	0.04
Absolute Reticulocyte Count (10³/µL)	17.33	15.73	14.83	15.47	5.829	0.48	0.47	0.49
Reticulocyte percentage (%)	0.27	0.27	0.24	0.23	0.094	0.25	0.79	0.20
Mean Reticulocyte Volume (fL)	44.87^ab^	48.98^a^	44.78^b^	45.68^ab^	1.113	0.13	0.04	0.05
Reticulocyte Cellular Hemoglobin (pg)	15.23^b^	17.02^a^	15.53^ab^	15.60^ab^	0.428	0.19	0.07	0.04
Reticulocyte Cellular Hemoglobin Concentration Mean (g/dL)	33.95^b^	34.63^ab^	34.77^ab^	34.15^ab^	0.215	0.67	0.78	0.04
Platelet count (10³/µL)	396.67	346	358	333.17	35.996	0.55	0.26	0.63
Plateletcrit (%)	0.26	0.24	0.25	0.23	0.025	0.85	0.31	0.76
Mean Platelet Volume (fL)	6.72^b^	6.77^b^	7.00^a^	6.98^a^	0.122	0.003	0.48	0.11
Large Platelets (%)	1.33^b^	1.83^a^	1.50^b^	2.17^a^	0.433	0.66	0.05	0.55
Mean Platelet Mass (pg)	1.51^b^	1.57^ab^	1.61^ab^	1.63^a^	0.031	0.02	0.20	0.03
Mean Platelet Component (g/dL)	24.13	24.72	24.38	24.75	0.249	0.72	0.17	0.28
Platelet Distribution Width (fL)	43.38^b^	46.12^a^	44.15^b^	47.73^a^	2.011	0.86	0.04	0.42
Platelet Mass Distribution Width (fL)	0.52^b^	0.57^a^	0.62^a^	0.62^a^	0.029	0.005	0.30	0.02
Platelet Component Distribution Width (fL)	6.53	6.48	6.28	6.3	0.117	0.09	0.74	0.34
7.5 h post LPS								
Red Blood Cell count (10^6^/µL)	6.62^a^	5.64^c^	6.10^b^	6.51^a^	0.202	0.42	0.20	0.01
Hematocrit (%)	28.38^a^	26.70^b^	26.33^b^	28.50^a^	0.763	0.88	0.76	0.03
Mean Corpuscular Volume (fL)	42.98	47.38	43.4	43.88	1.769	0.42	0.21	0.31
Macrocyte (%)	0.85	2.35	1.53	1.05	0.543	0.60	0.39	0.11
Hemoglobin (g/dL)	10.38^a^	9.58^b^	9.53^b^	10.45^a^	0.327	0.96	0.87	0.03
Mean Corpuscular Hemoglobin (pg)	15.73	17.03	15.7	16.08	0.689	0.51	0.27	0.53
Mean Corpuscular Hemoglobin Concentration (g/dL)	36.58	35.9	36.17	36.63	0.335	0.66	0.76	0.13
Cellular Hemoglobin (pg)	14.98	16.45	15.17	15.23	0.681	0.48	0.30	0.34
Cellular Hemoglobin Concentration Mean (g/dL)	34.75	34.55	34.77	34.55	0.22	0.97	0.38	0.97
Hypochromic RBC (%)	0.88	0.93	0.77	0.88	0.181	0.68	0.68	0.88
Hyperchromic RBC (%)	0.98	0.58	0.73	0.75	0.101	0.76	0.10	0.07
Red Cell Distribution Width (%)	17.73	17.2	18.7	17.78	0.6	0.24	0.27	0.75
Hemoglobin Distribution Width (g/dL)	2.4	2.25	2.32	2.34	0.053	0.95	0.26	0.15
Absolute Reticulocyte Count (10³/µL)	18.78	14.83	14.7	17.93	5.25	0.93	0.95	0.52
Reticulocyte percentage (%)	0.28	0.27	0.24	0.28	0.083	0.89	0.85	0.74
Mean Reticulocyte Volume (fL)	44.93	49.68	44.67	45.6	1.555	0.21	0.11	0.26
Reticulocyte Cellular Hemoglobin (pg)	15.55	17.03	15.27	15.7	0.633	0.25	0.17	0.45
Reticulocyte Cellular Hemoglobin Concentration Mean (g/dL)	34.45	34.5	34.27	34.53	0.368	0.84	0.69	0.79
Platelet count (10³/µL)	353.5	334.5	371.33	321.75	46.982	0.96	0.50	0.76
Plateletcrit (%)	0.24	0.24	0.26	0.24	0.033	0.76	0.61	0.76
Mean Platelet Volume (fL)	6.85^b^	7.00^b^	7.13^a^	7.23^a^	0.111	0.05	0.32	0.81
Large Platelets (%)	1.25	2.75	1.67	2.25	0.542	0.94	0.09	0.43
Mean Platelet Mass (pg)	1.54	1.61	1.66	1.62	0.04	0.12	0.69	0.19
Mean Platelet Component (g/dL)	24	24.63	24.63	24.15	0.566	0.90	0.91	0.37
Platelet Distribution Width (fL)	46.18	50.43	44.5	49.33	2.676	0.63	0.13	0.92
Platelet Mass Distribution Width (fL)	0.57	0.61	0.63	0.63	0.033	0.24	0.62	0.52
Platelet Component Distribution Width (fL)	6.45	6.45	6.1	6.5	0.113	0.23	0.12	0.12

Blood samples were collected at 5 and 7.5 hours after the intra-mammary LPS administration. Complete blood count analysis was performed at the hematology laboratory of the Veterinary Hospital of the Hebrew University (Rishon LeZion, Israel). Means with different superscript letters differ significantly (*P* ≤ 0.05). SEM, Standard error of mean. CTL, Control diet; BCP, β-Caryophyllene supplemented diet; CS, Cooling sessions per day (3 or 6) LPS, Lipopolysaccharide.

## 4 Discussion

In the present research, we investigated the effects of *in vivo* supplementation with a dietary CB2 ligand, BCP, to dairy cows with different CS frequencies during environmental heat load, and examined their performance, behavior and welfare parameters, and immune responses to an intramammary LPS challenge. Overall, we found that oral supplementation with BCP at 5 mg/kg BW was bioavailable, which is in line with a low ruminal degradation of BCP in the ovine model (31). Hence, we assume that the BCP dose used was sufficient to achieve systemic exposure and potentially induce CB2-mediated responses in immune cells (21). In the following sections we will discuss the effects of BCP supplementation, CS frequency and their interactions in cows during heat load and specifically focus on their immune response to an intra-mammary inflammatory challenge.

### 4.1 Effects of BCP supplementation and CS frequency on milk production, intake and eating behavior

Oral BCP supplementation at 5 mg/kg BW for 4 weeks did not affect DMI, milk yields, milk composition, or SCC in heat-stressed dairy cows. These findings are in line with others (32) that reported no effects of terpene supplementation on milk production and composition in dairy ewes, and with a report (33) that supplementing Holstein cows with a terpene blend containing BCP had no effect on DMI or milk production. In a recent study, Irawan et al. (2024) (34) fed lactating dairy cows with spent hemp biomass containing plant cannabinoids for 4 weeks and observed no effect on performance or on the immune system, although a slight low-grade inflammation and reduced liver clearance were suggested. Together, it seems that dietary cannabinoid-type compounds at moderate doses do not impair productive parameters in lactating dairy cows. Nevertheless, we found that eating time was reduced by 19.9% in BCP-supplemented cows compared to controls, without affecting DM intake, which may indicate that BCP affected feeding behavior patterns; however, we did not measure meal frequency or feed palatability in the present study. In contrast, others demonstrated that providing essential oil blends containing terpene compounds to dairy cows reduced DMI, potentially due to palatability concerns (35); however, the formulations and doses differed from the present study, and we did not observe intake suppression. In our previous study, mid-lactation cows during heat load were provided with a supplement containing plant extracts of green tea, red chili pepper and fenugreek, and their DMI was higher compared to controls (36). Overall, it was proposed that the effects of plant extracts supplementation on DMI and production in dairy cows are variable, and in most cases the supplementation does not have a pronounced effect on these parameters (37), which agrees with our present findings.

When comparing cooling regimens across dietary treatments, cows receiving 6CS had higher milk lactose concentrations, longer rumination times, and lower panting time and respiratory rates compared to cows receiving 3CS, which is in line with previous reports showing that increased cooling frequency alleviates the depression of intake and production caused by heat load (1, 38). The interaction of BCP × CS was not significant for most of the production, DMI and eating behavior parameters, suggesting that BCP supplementation did not have synergistic or antagonistic interactions with CS on these variables.

### 4.2 Effects of BCP supplementation and cooling frequency on resting behavior, cortisol, and their connection to stress and fever response

Among the most consistent behavioral effects of BCP supplementation in the present study was an increase in the duration of resting bouts, with a significant BCP × CS interaction for resting time, whereas the average daily resting time was not affected by BCP as a main effect. Cows in BCP-3CS had longer resting time than cows in CTL-3CS and did not differ from cows in CTL-6CS, which may indicate that BCP supplementation compensated, at least in part, for the lower cooling frequency. In addition, although plasma cortisol concentrations during the LPS challenge were not affected by BCP as a main effect, a tendency for lower cortisol AUC was observed in the BCP-6CS group compared to CTL-3CS. We propose that the longer resting bouts during the experiment and the tendency for a lower cortisol response during inflammatory challenge may reflect a BCP/CB2-mediated modulation of the stress response axis. In rodents, BCP was shown to elicit anxiolytic and stress-mitigating effects through CB2-dependent mechanisms, reducing anxiety-like behaviors and attenuating hypothalamic-pituitary-adrenal (HPA) axis activation (39). Furthermore, BCP administration reduced anxiety-like behavior and improved welfare-related behavioral parameters in mice through CB2-mediated pathways (40). In dairy cows, resting time is a well-established indicator of comfort and welfare; as prolonged lying time is associated with improved welfare scores, particularly under conditions of thermal and physiological stress (41). Therefore, the increase in duration of resting bouts we observed may indicate that the CB2-mediated responses by its specific agonist BCP could reduce stress-related behaviors, enabling longer rest periods. The tendency for lower cortisol concentrations in BCP-supplemented cows during the LPS challenge further supports this interpretation, suggesting that BCP may partially attenuate HPA axis activity (39).

In the present study, BCP-supplemented cows had lower peak fever response to the intra-mammary LPS challenge, indicating an anti-inflammatory effect. There could be a potential mechanistic link between the tendency for a reduced cortisol response and the lower peak vaginal temperatures observed in BCP-supplemented cows at 4.5–7 h post-LPS, as the tendency for attenuated cortisol secretion in BCP cows could indicate of a dampening of central inflammatory signaling that, in parallel, contributes to the reduction of peak fever observed in the vaginal temperature data. Since cortisol exerts mostly immune-suppressing or anti-inflammatory effects, this decrease would be expected to be associated with increased, rather than decreased, inflammatory signaling. As this was not observed, we propose two possible explanations: either cortisol was not a major mediator of the immunomodulatory effects of BCP, or the attenuated inflammatory response in BCP-supplemented cows reduced the physiological requirement for a compensatory anti-inflammatory signal such as cortisol.

Across dietary treatments, we found that cows in 6CS had higher resting time than cows in 3CS, which is in line with others that demonstrated that increasing the frequency of CS improves welfare parameters in dairy cows. For instance, increasing cooling frequency lowered cows’ rectal temperatures, and they spent more time lying down, indicative of improved welfare (38). The interaction between BCP × CS for resting time was significant, indicating a possible added effect of BCP supplementation and increased CS frequency on resting time. Together, these findings indicate moderate stress-mitigating and welfare-improving properties of BCP, along with the benefits of increased CS frequencies in heat-stressed dairy cows.

### 4.3 Effects of BCP supplementation on the inflammatory response to intramammary LPS challenge

During the LPS challenge, BCP-supplemented cows exhibited 0.45 °C lower vaginal temperatures at peak fever and a reduced temperature AUC during the 10 h post-LPS compared to the control cows. This intriguing effect of dietary BCP supplementation on inflammatory responses *in vivo* is unique and suggests of CB2-mediated effects. The mechanism of action of BCP is well characterized at the molecular level: BCP selectively binds to the CB2 receptor, which activates a G inhibitory protein signaling pathway, that inhibits adenylyl cyclase activity and reduces the intracellular production of cyclic adenosine monophosphate (cAMP), thereby attenuating the downstream activation of transcription factors that drive inflammatory gene expression (21). In dairy cows, we have recently demonstrated that *in vitro* supplementation of BCP to PBMC decreased the expression of several inflammatory genes following an LPS-challenge and tended to decrease the levels of cAMP in the medium of immune cells, which may indicate CB2-mediated responses and immune-modulating properties (22). In the landmark study by Gertsch et al. (2008) (21), BCP at 500 nM inhibited LPS-induced inflammatory responses in macrophages by suppressing phosphorylation of the MAP kinases ERK1/2 and JNK1/2, and BCP at 5 mg/kg BW reduced carrageenan-induced edema in wild-type but not CB2-deficient mice, demonstrating CB2-dependent anti-inflammatory effects *in vivo*. Recently, Bader Eddin et al. (2026) (42) reviewed the molecular and pharmacological actions of BCP and emphasized that, beyond CB2 activation, BCP also interacts with peroxisome proliferator-activated receptors (PPAR) nuclear receptors, and modulates the AMPK signaling pathway, contributing to its anti-inflammatory, antioxidant, and immunomodulatory effects across several tissues.

In the present study, we did not evaluate directly CB2 activation, however we assume that the BCP exerted CB2-mediated responses. Mechanistically, the fever response during LPS challenge is mediated by the sequential release of pro-inflammatory cytokines (such as TNFα, IL-1β, IL-6) from activated immune cells, that stimulate prostaglandin E2 (PGE2) production in the hypothalamus via cyclooxygenase-2 (COX-2); PGE2 then acts on hypothalamic thermoregulatory neurons to elevate body temperature (43). We propose that CB2-mediated responses by BCP may interrupt this cascade at several levels, including reduction of cytokine secretion from circulating immune cells (21) and attenuation of PGE2 production. Moreover, the ECS is also directly involved in thermoregulation, as the endocannabinoid 2-AG is hydrolyzed to AA by monoacylglycerol lipase (MGLL), providing substrate for COX-2-mediated PGE2 synthesis (44, 45); therefore, modulation of the ECS by BCP-mediated CB2 signaling may further modulate eicosanoid-driven fever; however, further studies should examine this hypothesis. Our main finding related to the immune-modulating effects of BCP is the reduction in fever response, which is a basic inflammatory response in mammals. However, we did not reach significance when measuring cytokines and chemokines concentrations in plasma, which limits our claim of systemic anti-inflammatory effects of BCP. The lack of significance in cytokine concentrations among the groups may be related to the variability between cows and suggests that a larger number of animals could provide better clarity on the effects of BCP on cytokine concentrations post-LPS.

Of particular interest, MIP-1β concentrations in plasma were reduced in BCP-supplemented cows compared to controls at 14.5 and 24 h post-LPS, and 6CS tended to reduce MIP-1β across the 24 h post-LPS compared to 3CS. MIP-1β is a pro-inflammatory chemokine primarily produced by activated monocytes and macrophages in response to bacterial products, and it acts as a chemotactic and adhesion factor that recruits monocytes, T cells, and other immune cells to sites of inflammation (46). The reduction in MIP-1β post-LPS may indicate attenuated monocyte activation following CB2 stimulation. This finding is of interest in the context of our previous *in vitro* study using bovine PBMC, where we demonstrated that *in vitro* heat stress increased LPS-induced MIP-1β secretion, while under thermoneutral conditions MIP-1β was not significantly affected by LPS or BCP (22). Together, these findings indicate that MIP-1β is a particularly stress- and CB2-sensitive chemokine in bovine immune cells, and that CB2-mediated responses may attenuate MIP-1β release *in vivo* under conditions of environmental heat load. In this study, both controls and BCP-supplemented cows had similar MDA levels post-LPS. BCP can have anti-oxidant effects (39), however we did not detect changes in MDA, thus we assume that the main effect of BCP was via CB2-mediated responses and propose that possibly a higher dose of BCP could have the potential to lead to changes in anti-oxidant pathways *in vivo*.

### 4.4 Effects of LPS and BCP on peripheral blood immune cell populations and hematological parameters

To characterize the cellular immune response to the LPS challenge, we used flow cytometry to quantify several main WBC subpopulations defined expression of cell type-specific surface markers expression. CD4+ cells are T helper lymphocytes that orchestrate adaptive immunity through cytokine secretion and activation of other immune cells (24), whereas CD8+ cells are cytotoxic T lymphocytes that, in addition to eliminating infected or transformed cells, also exert regulatory and cytokine-secreting functions during infections with extracellular pathogens such as Gram-negative bacteria (25). Monocytes constitute a heterogeneous population of phagocytic cells that contribute to innate immune responses and tissue repair; they can be classified according to their expression levels of CD14 and CD16 (26). In cattle, these markers are particularly informative, as the functional capacities of CD14+CD16− (classical), CD14+CD16+ (intermediate), and CD14−CD16+ (non-classical) subsets differ markedly (26, 27). CD14 is a co-receptor for LPS that helps transfer/present LPS to the toll-like receptor 4 (TLR4), and is highly expressed on monocytes (26). CD16 is a marker of non-classical monocytes and NK cells, and CD14+CD16+ cells represent an intermediate monocyte subset with enhanced pro-inflammatory cytokine secretion capacity (28). In the present study, a marked decrease in the percentages of CD14+, CD16+, and CD14+CD16+ double-positive cells was observed at 5 h post-LPS compared to pre-LPS, alongside an increase in CD4+ T helper cells. The pronounced reduction in circulating CD14+ and CD16+ populations at 5 h post-LPS likely represents a transient migration of classical, intermediate, and non-classical monocyte subsets out of circulation toward the LPS-challenged mammary quarter, where these cells contribute to bacterial clearance through phagocytosis and cytokine release (26). These results are in agreement with Thaler et al. (2016) (26), that reported a drop in CD14+ and CD16+ monocytes in human blood following an intravenous LPS injection. This interpretation is further strengthened by our observation that the total number of WBC recovered at 5 h post-LPS was notably lower than pre-LPS, consistent with cellular migration out of circulation into the inflamed tissue.

CD335 is a natural cytotoxicity receptor that marks NK cells in cattle (29), and WC1 is a scavenger receptor expressed specifically on a major subpopulation of γδ T cells in ruminants (30). The BCP × CS interactions detected for CD16+, CD335+, WC1+, and CD335+WC1+ cells after LPS indicate that the combination of CB2-mediated responses and the intensity of cooling sessions reshape both innate (monocyte, NK) and γδ T cell responses to the LPS challenge.

In the CBC analysis, we found that monocyte percentage was lower at 5 h compared to 7.5 h post-LPS. The lower percentage at 5 h could indicate of migration of monocytes to the inflamed tissue; therefore, the increase in 7.5 h may suggest a beginning of inflammation resolution and a resume to basal levels of circulating monocytes, as can also be concluded by the decrease in cytokine/chemokine concentrations at these times post LPS. Activation of the CB2 receptor in human monocytes has been shown to reduce the release of pro-inflammatory cytokines such as TNFα, IL-1β and IL-6 after stimulation by LPS *in vitro*, although it has not been shown that CB2 activation reduces the percentage of monocytes (47). However, in our study the lack of differences in absolute monocyte counts between treatments suggests that these percentage shifts may also reflect changes in the relative abundance of other WBC subpopulations rather than direct modulation of monocyte numbers. Additionally, eosinophil percentages at 5 h post-LPS were higher in 6CS than in 3CS groups, and basophil percentages tended to be higher in BCP-supplemented cows at both 5 h and 7.5 h post-LPS. Yoon et al. (2019) (48) showed that eosinophils are activated through TLR4 and, *in vitro*, secrete cytokines and chemokines such as IL-1β, IL-6, IP-10 and MIP-1α upon LPS stimulation, that could indicate of an early eosinophil contribution to the cytokine peak observed at this time-point.

Within the platelet panel of the CBC at 5 h post-LPS, BCP supplementation was associated with higher mean platelet volume (MPV), increased mean platelet mass and platelet mass distribution width, while platelet component distribution width tended to be reduced by BCP. At 7.5 h post-LPS, MPV remained higher in BCP-cows with a tendency toward significance compared to CTL. Platelets are recognized as active participants in the regulation of inflammatory responses through interactions with leukocytes, the vasculature, and immune signaling molecules; larger platelets are generally considered more metabolically active and contain greater quantities of pro-inflammatory and pro-resolving granule contents (49). Although we did not assess directly the functionality of platelets in the present study, the increase in MPV and platelet mass in BCP-supplemented cows post-LPS could indicate enhanced platelet activation and functional engagement in the immunological response to the mammary challenge (49). Moreover, the changes in platelet indices in BCP-supplemented cows correlate with the changes in circulating monocyte percentages and basophil counts during the 24 h post-LPS, indicating a coordinated hematological response that may be partially mediated by CB2-related responses. Thus, BCP may exert specific moderate immunomodulatory effects on both circulating immune cells and platelets during an acute inflammatory challenge in dairy cows.

## 5 Conclusions

In the present research, we found that BCP supplemented at 5 mg/kg BW was bioavailable and did not affect performance in lactating cows. Cows supplemented with BCP had some moderate immune-modulating and stress-mitigating properties that may reflect a distinct CB2-mediated modulation of the immune and stress responses. The significant BCP × CS interactions detected for resting behavior and specific immune parameters suggest that the combination of BCP and frequent CS may also affect the response to heat load and inflammation. Mostly based on our intriguing finding of lower fever response to an intra-mammary LPS challenge, we propose that dietary BCP, and more generally CB2 agonists, may have moderate anti-inflammatory properties in heat-stressed dairy cows, and could serve as a novel nutritional strategy under environmental heat load. Further studies should investigate the mechanisms underlying these effects and validate them in cattle experiencing various inflammatory states.

## Data Availability

Data sharing will be considered by the authors upon request. Requests to access the datasets should be directed to Maya Zachut, mayak@volcani.agri.gov.il.
