# Supplementary material for: Immune-modulating effects of dietary cannabinoid-receptor 2 agonist beta-caryophyllene and cooling frequency in dairy cows under environmental heat load
Source: Front Immunol. 2026 Sep 30;17:1931588. doi: 10.3389/fimmu.2026.1931588 (PMC13661458; doi:10.3389/fimmu.2026.1931588)
Supplement: Supplementary file 1 [file Table1.docx]

Supplementary materials – Hubner et al., 2026

**Supplementary Figure 1**. Example of gating strategy, shown on samples from cow #4150 (BCP-6CS) after LPS challenge. A) Fluorescence-minus-one (FMO) or unstained control plots used to set positivity thresholds (A647/RPE and A647/FITC), gated on the ungated total events (10,000 events/plot); quadrant gates were set based on background/negative staining and applied uniformly across corresponding stained samples. B) Representative dot plots of stained samples showing positive and negative populations for CD4/CD8 (T-cell subsets), CD14/CD16 (monocyte subsets), and CD335/WC1 (NK cell/γδ T-cell subsets), with the percentage of cells falling within each quadrant indicated. A647 = Alexa Fluor 647; FITC = fluorescein isothiocyanate; RPE = R-phycoerythrin.
